# RETRACTION: High‐Frequency Ultrasound Evaluation of Morphea: Retrospective Analytical Study

**DOI:** 10.1111/srt.70320

**Published:** 2026-01-10

**RETRACTION**: T. Yazdanparast, A. Mohseni, K. S. Dehghan, S. Delavar, and A. Firooz, “High‐Frequency Ultrasound Evaluation of Morphea: Retrospective Analytical Study,” *Skin Research and Technology* 30, no. 7 (2024): e13818, https://doi.org/10.1111/srt.13818.

The above article, published online on 25 June 2024 in Wiley Online Library (wileyonlinelibrary.com), has been retracted by agreement between the authors; *Skin Research and Technology*; and John Wiley & Sons Ltd. The article was accepted for publication following an insufficient peer review process that does not meet the standards of the journal's peer review policy. Upon further review, it was noted that the conclusions presented in the article are not supported by the data. Although the manuscript acknowledges the absence of statistically significant differences, it nonetheless concludes that morphea lesions exhibit increased dermal thickness and reduced dermal density compared to control regions as measured by high‐frequency ultrasound (HF‐US), thereby supporting the validity of the methodology in the assessment of morphea disease.

At the journal's request, the authors provided the full set of raw data underlying the study. A review of the patient‐level data revealed that these trends are inconsistent across the cohort and appear to be spurious, indicating potential misinterpretation of the aggregated data. The authors maintain that these trends were confirmed by histopathology, as stated in the article. However, the conclusions regarding the validity of HF‐US in assessing morphea disease through measurements of dermal thickness and density remain unsupported. Accordingly, the article has been retracted.

